# Academic and Community Hospitals differ in stroke subtype classification and cardiac monitoring: the DiVERT multi-center cohort study

**DOI:** 10.3389/fneur.2024.1428731

**Published:** 2024-12-04

**Authors:** David Z. Rose, Ruchir A. Shah, Josh Snavely, Marla Hairston, Stephen Adams, Paul D. Ziegler, Sarah C. Rosemas, Michael Chandler, Roberto Carta, Karah B. Neisen, Noreli C. Franco, Thomas G. Devlin

**Affiliations:** ^1^Department of Neurology, University of South Florida Morsani College of Medicine, Tampa, FL, United States; ^2^CHI Memorial Neuroscience Institute, Common Spirit Health System, Chattanooga, TN, United States; ^3^Franciscan Neurology Associates, Virginia Mason Franciscan Health, Tacoma, WA, United States; ^4^Medtronic, Inc., Cardiac Rhythm Management, Mounds View, MN, United States

**Keywords:** acute ischemic stroke, cardiac rhythm monitoring, atrial fibrillation, anticoagulation, care pathway

## Abstract

**Background:**

Cardiac monitoring strategies to detect occult atrial fibrillation (AF) post-stroke differ among healthcare institutions. This may be related to discrepancies in stroke subtype classification/adjudication, and/or consultation of cardiology specialists at Community Hospitals (CoH) and Academic Centers (AcC). Identifying the degree of heterogeneity may encourage development of guideline-directed monitoring protocols, result in higher AF detection rates and treatments, and fewer strokes.

**Methods:**

The DiVERT (SeconDary Stroke PreVEntion ThRough Pathway ManagemenT) study was designed to characterize post-stroke cardiac monitoring practices in a hospital setting. Care pathways were assessed with in-person stakeholder interviews; patient-level data were reviewed using electronic medical records.

**Results:**

DiVERT identified 2,475 patients with diagnoses of cryptogenic (83.6% vs. 33.1%, *p* < 0.001), large vessel disease (LVD) (13.3% vs. 37.0%, *p* < 0.001), or small vessel disease (SVD) (3.1% vs. 29.9%, *p* < 0.001) stroke, at CoH and AcC, respectively. CoH consulted cardiology significantly less than AcC (12.3% vs. 34.7%, *p* < 0.001) and ordered significantly fewer short- or long-term cardiac monitors than AcC (6.8% vs. 69.2%, *p* < 0.001). CoH had shorter length of stay (5.3 vs. 9.4 days, *p* < 0.001) and patient demographics were significantly different (*p* < 0.001 for age, ethnicity and race).

**Conclusion:**

Significant heterogeneity in cardiac monitoring post-stroke exists: CoH reported 2.5-times more cryptogenic stroke than AcC yet ordered 10-times fewer short/long-term cardiac monitors to look for AF. Significant differences in patient demographics among institutions may account for this discrepancy. Regardless, efforts to reduce heterogeneity are warranted to improve AF detection and treatment and prevent recurrent stroke.

## 1 Introduction

Newer-generation cardiac monitoring devices to detect occult/paroxysmal atrial fibrillation (AF) post-stroke, such as mobile cardiac telemetry and insertable cardiac monitors (ICMs), have led to improved outcomes in multiple clinical trials over the past decade (1, 2). Higher AF detection rates result in fewer ischemic strokes because they lead to further appropriate treatment such as anticoagulation instead of antiplatelet therapy (1). AF-related strokes result in larger-volume brain infarctions, longer hospital stay, double in-hospital complications, morbidity and mortality, and AF poses a 5-fold higher risk of stroke than in those without AF (3, 4). Therefore, earlier detection of AF has significant clinical and economic ramifications—even in patients unsuitable for anticoagulation, because non-pharmacologic options exist, such as endovascular or surgical left atrial appendage (LAA) closure (4–6). Subsequently, guideline updates in the United States and Europe support long-term rhythm monitoring with mobile cardiac telemetry or ICM post-stroke (class IIa) (7–10). However, multiple obstacles have prevented their use at various healthcare institutions (11). Whether such barriers occur primarily at Community Hospitals (CoH) or Academic Centers (AcC) is unknown. Whether these barriers impede the use of external/short-term monitors or long-term ICMs is also unknown.

The DiVERT (SeconDary Stroke PreVEntion ThRough Pathway ManagemenT) study characterized cardiac monitoring practices post-stroke at CoH and AcC for cryptogenic, large-vessel disease (LVD), and small-vessel disease (SVD) stroke patients. The rationale for this was to uncover the degree of heterogeneity among both large and small, public and private, urban, suburban and rural centers to maximize generalizability of a wide array of stroke patient demographics. Based on our findings, we will design an assessment to further research potential solutions and evaluate their impact on clinical stroke outcomes with the aim of curating consistent, guideline-directed monitoring protocols to improve AF detection, initiate AF treatment (anticoagulation and/or LAA management) and prevent recurrent strokes.

## 2 Methods

### 2.1 Design

DiVERT was a retrospective, non-randomized, multi-center, nationwide cohort study across the United States, designed to evaluate care pathways for patients with cryptogenic, LVD, or SVD stroke in the inpatient hospital setting (Clinical Trial Registration: ISRCTN87407792).^1^ The DiVERT study was approved by all participating study hospital Institutional Review Boards. All study procedures were in accordance with the provisions of the International Conference of Harmonization Good Clinical Practice and the 1964 Declaration of Helsinki and its later amendments. Due to the retrospective nature of this study, patients were not required to sign an Informed Consent Form; waiver of Informed Consent was obtained from the Institutional Review Board (IRB) at each site.

The specific aim of this study was to collect and analyze post-stroke pathway data from both AcC and CoH and identify the degree of heterogeneity among these centers. Patients were retrospectively identified by 8 separate clinical study sites in Florida, Tennessee, South Carolina, Texas, and Washington state (see Supplemental material 1). All participating centers provided the study sponsor with: (1) documentation of historical (January 1, 2017 to July 31, 2019) and current stroke care pathways (as of December 31, 2019), standard order sets, and other protocols related to stroke patient care; (2) de-identified patient-level data from medical records; and (3) access to linked patient-level data from the American Heart Association’s Get With The Guideline (GWTG) hospital-based quality improvement program. These items delivered a longitudinal view of patient care and outcomes. Data extraction and data linking were performed by site-specific personnel.

### 2.2 Patient population, inclusion, and exclusion criteria

Eligible patients were 18 years of age or older and had an index stroke classified as cryptogenic, LVD, or SVD (12). Cryptogenic stroke was defined individually at each institution. The cryptogenic designation typically required a minimum workup including routine stroke laboratory studies, brain MRI, transthoracic echocardiography, cervical and intracranial vascular imaging, admission EKG and cardiac telemetry while hospitalized. Additional tests were performed during the initial hospitalization when clinically warranted according to the discretion of the treating neurologist. Designation of the stroke as “cryptogenic” in the GWTG database was typically based on the stroke etiology deemed most appropriate at the time of hospital discharge.

### 2.3 Data collection and study outcomes

Data collection was performed on linked and de-identified patient-level data to enable definition and analysis of the stroke care pathway, variation in care and outcomes, statistical methodology, and opportunities for improvement. Qualitative assessment and analysis of existing stroke care pathways were conducted at each study site, focusing on stroke care pathways starting at hospital admission (day 0) and through 180 days post-discharge. Interviews and meetings with clinicians and staff identified type and extent of stroke care pathways. Past medical history was collected within 12 months prior to the index stroke hospitalization. During index hospitalization, dates (admission and discharge), neurological diagnostic testing, cardiology consultation, and use of short-term/external monitors and/or ICM were collected. Cardiac monitoring data was collected from hospital admission through 180 days of follow-up. For follow-up visits through 180 days post-discharge, discharge location (skilled nursing facility, inpatient rehabilitation, home, etc.), mortality, recurrent stroke, and cardiovascular disease-related healthcare encounters were also collected. Results were provided back to each clinical study site for open discussion and to help generate hypotheses around potential areas for improvement.

### 2.4 Statistical analyses

This analysis was presented in adherence with the STROBE statement (13). Analyses included all eligible patients with cryptogenic, LVD or SVD stroke hospitalization at each study site. Index stroke hospitalization was defined as first hospitalization due to stroke during the follow-up period (2017–2019). This pre-pandemic period was chosen to avoid confounders and comorbidities introduced into the healthcare system because of COVID-19. No goal sample size was targeted for the analyses; all patients that fit inclusion and exclusion criteria at their respective study sites contributed to the final data pool and to the final population size. Exploratory data analysis was carried out and descriptive statistics were obtained for basic patient demographics, relevant performance indicators and clinical observations. Tests of equality of means and proportions were performed using t-tests and contingency table methods using chi-squared tests. No adjustment for multiple comparisons was done as the analysis was exploratory in nature. Any data that could not be extracted were not included in the final analyses.

## 3 Results

**Figure 1:**
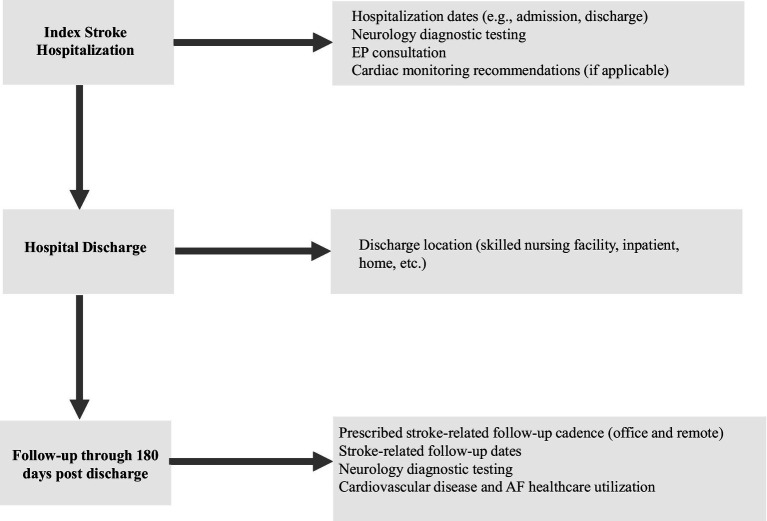
DiVERT study flowchart.

The DiVERT study flowchart is shown in Figure 1. A total of 2,475 stroke patients were identified and included in the analyses: 1,783 from CoH and 692 from AcC. Table 1 shows significant differences for age (70.3 vs. 64.6 years, *p* < 0.001), race (75.3% vs. 63.2% White; 9.3% vs. 25.0% Black, *p* < 0.001), and ethnicity (3.2% vs. 11.0% Hispanic, *p* < 0.001) between CoH and AcC, respectively, while sex was similar for both groups (52.2% vs. 55.1% male, *p* = 0.19).

**Table 1:** Baseline demographics.

	Total (*N* = 2,475)	CoH (*n* = 1,783)	AcC (*n* = 692)	*P*-value
**Age (years) Mean (SD)**	68.7 (13.6)	70.3 (13.0)	64.6 (14.4)	<0.001
**Male, n (%)**	1,311 (53.0)	930 (52.2)	381 (55.1)	0.19
**Race**				<0.001
White	1,779 (71.9)	1,342 (75.3)	437 (63.2)	
Black	339 (13.7)	166 (9.3)	173 (25.0)	
Asian	145 (5.9)	126 (7.1)	19 (2.7)	
American Indian	16 (<1.0)	14 (<1.0)	2 (<1.0)	
Native Hawaiian or Pacific Islander	50 (2.0)	50 (2.8)	0 (0.0)	
Other	146 (5.9)	85 (4.8)	61 (8.8)	
**Ethnicity**				<0.001
Hispanic	133 (5.4)	57 (3.2)	76 (11.0)	
Not Hispanic or unknown	2,342 (94.6)	1,726 (96.8)	616 (89.0)	
**Age Group (years), n (%)**				<0.001
<50	218 (8.8)	111 (6.2)	107 (15.5)	
50–74	1,367 (55.2)	968 (54.3)	399 (57.7)	
>75	890 (36.0)	704 (39.5)	186 (26.9)	

Stroke subtype adjudications (Table 2) were significantly different between CoH and AcC: cryptogenic stroke was diagnosed in 83.6% vs. 33.1% (*p* < 0.001), LVD in 13.3% vs. 37.0% (*p* < 0.001) and SVD stroke in 3.1% vs. 29.9% (*p* < 0.001) patients, respectively.

**Table 2:** Ischemic stroke subtypes.

	Total (*N* = 2,475)	CoH (*n* = 1,783)	AcC (*n* = 692)	*P*-value
**Subtype**				<0.001
Cryptogenic	1,719 (69.5)	1,490 (83.6)	229 (33.1)	
LVD	493 (19.9)	237 (13.3)	256 (37.0)	
SVD	263 (10.6)	56 (3.1)	207 (29.9)	

Differences in hospitalization resource utilization were also observed (Table 3). CoH were significantly less likely than AcC to consult Cardiology during index stroke hospitalization (12.3% vs. 34.7%, *p* < 0.001). Length-of-stay was significantly shorter at CoH than AcC (5.3 vs. 9.4 days, *p* < 0.001), however, reasons contributing to extended hospital stay were not available.

**Table 3:** Hospitalization resource utilization.

	Total (*N* = 2,475)	CoH (*n* = 1,783)	AcC (*n* = 692)	*P*-value
Cardiology consultation during index event	460 (18.6)	220 (12.3)	240 (34.7)	<0.001
Length-of-stay (days)	6.5 (9.0)	5.3 (7.0)	9.4 (12.4)	<0.001

Data collected starting at hospital admission and through 180 days post-discharge (Table 4) revealed that CoH ordered significantly fewer cardiac monitors than AcC: external/short-term monitor in 5.3% vs. 65.5% (*p* < 0.001); ICM in 1.6% vs. 10.5% (*p* < 0.001); any cardiac monitoring in 6.8% vs. 69.2% (*p* < 0.001). Cardiovascular disease-related healthcare encounters were similar at CoH and AcC (19.3% vs. 19.8%, *p* = 0.80), and recurrent stroke rate was lower in CoH than AcC (5.1% vs. 9.1%, *p* < 0.001).

**Table 4:** Data collected from admission through 180 days post-discharge.

	Total (*N* = 2,475)	CoH (*n* = 1,783)	AcC (*n* = 692)	*P*-value
Any cardiac monitoring	600 (24.2)	121 (6.8)	479 (69.2)	<0.001
External monitor	547 (22.1)	94 (5.3)	453 (65.5)	<0.001
ICM	102 (4.1)	29 (1.6)	73 (10.5)	<0.001
Cardiovascular disease-related healthcare encounters	482 (19.5)	345 (19.3)	137 (19.8)	0.80
Recurrent stroke	154 (6.2)	91 (5.1)	63 (9.1)	<0.001

## 4 Discussion

This study has unearthed substantial heterogeneity of cardiac monitoring practices at community and academic hospitals after acute ischemic stroke of cryptogenic, SVD, or LVD etiology. Considerable differences were identified in stroke subtype adjudication, cardiology consultation and extended cardiac monitoring. At CoH, 12.4-fold fewer external/short-term monitors and 6.6-fold fewer ICMs were placed compared to AcC—overall 10.2-fold less monitoring with either a short- or long-term device by CoH—despite a reported 83.6% cryptogenic stroke rate at CoH. Prior studies have alleged that underuse of cardiac monitoring has likely caused a population-wide over-diagnosis of cryptogenic strokes and simultaneous under-diagnosis of AF (11, 14). This is congruent with the 2.5-fold greater diagnosis of cryptogenic stroke at CoH vs. AcC, 2.8-fold greater likelihood of diagnosing LVD stroke, and 9.6-fold greater likelihood of diagnosing SVD stroke.

A difference in 4.1-day longer length-of-stay (LOS) at AcC than CoH was observed, although the reasons leading to this difference were not collected. However, we could speculate that with over 200 known causes of ischemic stroke (15), it is possible that a deeper dive into the workup during stroke admission at AcC may have resulted in longer hospitalizations at these centers. Earlier discharge and shorter length of stay (LOS) at CoH could also be explained by providers at CoH who shift workup to outpatient instead of inpatient, which may be more cost effective and user-friendly for the patient. However, if this were the cause, this diagnostic deferral could prevent clear etiological identification of the stroke at discharge and hence incomplete or inaccurate adjudication for GWTG. Longer LOS could also be explained by more complex cases arriving to AcC than to CoH—a byproduct of how the healthcare system is set up in the United States with geographical and insurance considerations. Expectedly, sicker patients require longer stay, but also vascular neurology subspecialists at AcC (not always available at CoH) may have requested more extensive inpatient workup, consultations, and more laboratory or radiographic tests than general neurologists at CoH. Indeed, at CoH, internists and non-vascular trained neurologists may be the principal providers to admit and manage stroke patients. This may also explain earlier discharge and shorter LOS at CoH, and if CoH lack a formalized Neuro-Cardio Program (NCP) program or pathway, it may also explain fewer requests for extended cardiac monitoring. Uniform, stroke-specific pathways can be utilized by providers of various specialties to standardize etiological workup. These can be built by vascular neurologists, but if they are unavailable in a particular area of the country (i.e., rural or underserved region), then the option of telestroke may help CoH improve the rates of TOAST classification. Telestroke is often staffed by stroke trained neurologists who are likely more inclined to identify etiologies/diagnoses for GWTG. Telestroke physicians may also be able to help CoH approach the expected rates of cryptogenic, SVD and LVD reported by AcC, as well as augment the ordering of cardiac monitors in those patients who need them.

Extended workups have become routine practice for patients with non-lacunar embolic stroke of undetermined source (ESUS), a subset of cryptogenic stroke with embolic-appearing neuroradiographic patterns (16). Indeed, the designation of “cryptogenic stroke” entered into GWTG is based on the initial inpatient hospital workup only—this can change after further workup (TEE, ICM, hypercoagulable tests, etc.) as an outpatient reveals an etiology. This may also partially explain the lopsided rates of cryptogenic strokes at CoH. While currently not collected in the GWTG database, collection of final stroke etiology during outpatient stroke workup would provide highly useful information on the quality of stroke care on a national level.

Cryptogenic/ESUS workup is typically a diagnosis of exclusion only after thoroughly searching for cardioembolic etiologies with TEE (15) and occult AF with ICM, among other tests (15, 17, 18). Based on CRYSTAL-AF, a longer-term monitoring strategy with ICM is superior for AF detection in cryptogenic/ESUS patients: the median time was 84 days from ICM insertion to AF discovery when patients were monitored for 12 months (18). This is most relevant for the highest-risk ESUS patients, such as those over 75 years of age (16, 19, 20).

Besides cryptogenic/ESUS patients, ICM is also superior for AF detection in SVD and LVD stroke, based on the STROKE AF trial (2, 17). Although these results were announced after the timeframe in which DiVERT explored, SVD and LVD subtypes were included in DiVERT to assess pre-STROKE AF heterogeneity of classification and monitoring, presuming that future practice patterns were likely to change. This also identified a baseline population of SVD and LVD patients in whom monitoring was performed even prior to the publication of STROKE AF results.

Because AF detection typically leads to anticoagulant initiation for stroke prevention (18), and because data have shown that AF may remain undetected for months post-stroke (18), it is reasonable to include ICM in care pathway protocols for cryptogenic, LVD and SVD stroke (20). Asking patients to return to clinic for ICM placement (after short-term monitoring post-discharge) is inconsistent and disappearance/recidivism is high (21). If monitoring had stopped at 30 days in CRYSTAL-AF, 88% of cryptogenic patients who eventually had AF detected by ICM would have remained cryptogenic (AF undiagnosed) (18). One study found that only 64% of stroke patients wore short-term monitors for the full-duration prescribed, 25% never wore them, and paradoxically, compliance with monitoring worsened as AF-stroke risk score increased (22).

Limitations in our study are: first, its observational nature, which makes it unable to draw comparative conclusions and to control variability among hospitals. Hence, baseline comorbidities and vascular risk factor scores (i.e., CHA_2_DS_2_-Vasc, HAS-BLED, NIHSS) were unable to be collected in a consistent manner across all study sites. Secondly, the CoH and AcC in this study are a relatively small sample of hospitals and only represent the Midwest, South, Southwest, Southeast, and Northwest regions of the US, limiting the generalizability of the findings as differences in patient demographics and hospital resources in unrepresented regions could result in different outcomes. Third, although TOAST criteria for stroke etiology determination have been utilized for decades, and provider-specific uncertainty exists, overall verity of data is called into question when only 3.1% of CoH patients were adjudicated (or perhaps, merely reported) as SVD stroke, and 83.6% were deemed cryptogenic. The designation of “cryptogenic” in the GWTG database where our results were extracted may have been erroneously entered by stroke coordinators or data abstractors not fully versed in proper stroke diagnosis adjudication based on chart review. Despite the specific factors at play, this finding is of concern as it points to errors that exist in the GWTG database which in turn greatly limits our ability to use it for quality patient care tracking. Implementation of new technologies to more directly allow experienced clinicians to enter correctly adjudicated stroke data into the national stroke databases will be critically important. Such technologies are now under development. DiVERT was an eye-opening, real-world, retrospective review with a widely generalizable, diverse population gathered from multiple institutions of various sizes.

The value-add of a hospital utilizing an official Neuro-Cardio Program cannot be overstated. Nearly 3-fold more cardiology consultations at AcC potentially led to fewer diagnoses of cryptogenic strokes compared to CoH. Involvement of cardiology in stroke care has been shown to improve both cardiologic and neurologic outcomes (10–12). Patients have lower future stroke risk if seen by cardiology versus primary care (HR 0.90; 95% CI, 0.86–0.94) (23). Oral anticoagulant prescriptions were more frequently dispensed with cardiology providers within 90 days of AF diagnosis (24, 25). Neurologist-cardiologist collaboration can include shared decision-making (SDM) discussions with patients, families and other providers, that for example, offer LAA closure as non-pharmacologic alternative to oral anticoagulant for AF patients with intracerebral hemorrhage, cerebral amyloid angiopathy/cerebral microbleeds, coagulopathies, hemophilia, thrombocytopenia, chemotherapy, planned surgery/dental procedures, advanced age, fall risk, and hazardous occupations (26). For stroke patients with AF, cardiologists can: adjust anti-arrhythmic and anti-hypertensive medications, statins for hyperlipidemia, and anti-platelets (single vs. dual) for coronary stents; assess for myocardial ischemia or left heart catheterization; and perform transthoracic and transesophageal echocardiograms (TEE) to evaluate ejection fractions, valvulopathies, patent foramen ovale/septal aneurysms, atrial/ventricular thrombi, septic/marantic endocarditis, fibroelastoma, myxoma, and more.

In particular, all the demographic data at AcC and CoH were significantly dissimilar, with the exception of sex, making DiVERT a comparison of different populations of stroke patients. Indeed, the lower recurrent stroke rate at CoH may reflect a sicker population at AcC, some of whom may have been referred from CoH to AcC for neurosurgical or neurointerventional needs unavailable at CoH. Moreover, patients at AcC were more than twice as likely as those at CoH to consult cardiology. This may also be similarly explained by a sicker population with more cardiological co-morbidities at AcC than CoH, resulting in patient referrals to AcC for cardio-thoracic surgical or cardio interventional needs unavailable at CoH. General neurologists may manage stroke patients at CoH (unless tele-stroke is available at that institution) compared to stroke-fellowship trained, subspecialty-boarded vascular neurologists at AcC. Similarly, while general cardiologists may answer consultations at CoH, fellowship trained, subspecialty-boarded electrophysiologists, structural heart specialists, and heart failure experts are typically available at AcC.

In summary, DiVERT Phase I established a baseline for post-acute stroke cardiac monitoring. We uncovered a Pandora’s Box of disconcerting major gaps in stroke care at both AcC and CoH. This underscores the urgent unmet need for more standardized, guideline-driven, post-stroke protocolization. With these data, future site-specific quality improvement and observational prospective research can focus on addressing rate-limiting steps, bottlenecks, and obstacles for cardiac monitoring. New, automated, AF-detection technologies are omnipresent, making it easier for clinicians to diagnose and treat quickly to prevent recurrent stroke. Pre-defined data dictionaries that automatically collect relevant stroke metrics in real-time are supplanting older, slower data collection methods, which only report inpatient assessment of etiology, if identified. DiVERT Phase I has shown that further research is needed to overcome barriers to cardiac monitoring, improve accuracy of longitudinal data capture, patient follow-up, and referral to cardiology providers. It is our intent that Phase II addresses these items.

## 5 Conclusion

DiVERT Phase I identified significant heterogeneity in stroke subtype classification and cardiac monitoring post-stroke: CoH reported 2.5-times more cryptogenic stroke than AcC yet ordered 10-times fewer short/long-term cardiac monitors to look for AF. These results suggest that guideline-directed monitoring protocols are needed to reduce heterogeneity, improve AF detection and treatment, and prevent recurrent stroke. DiVERT Phase II will attempt to address these discrepancies.

## Data Availability

The datasets presented in this article are not readily available because data was collected under a clinical study. Requests to access the datasets should be directed to karah.neisen@medtronic.com.

## Footnotes

^1^ https://www.isrctn.com/ISRCTN87407792
