# Supplementary material for: Academic and Community Hospitals differ in stroke subtype classification and cardiac monitoring: the DiVERT multi-center cohort study
Source: Front Neurol. 2024 Dec 4;15:1428731. doi: 10.3389/fneur.2024.1428731 (PMC11652527; doi:10.3389/fneur.2024.1428731)
Supplement: Supplementary file 1 [file Data_Sheet_1.pdf]

List of Participating Clinical Study Sites

CHI Memorial Hospital, Chattanooga, TN

St. Joseph Medical Center, Tacoma, WA

St. Michael Medical Center, Silverdale, WA

St. Clare Hospital, Lakewood, WA

St. Francis Hospital, Federal Way, WA

St. Anne Hospital, Burien, WA

St. Anthony Hospital, Gig Harbor, WA

University South Florida / Tampa General Hospital, Tampa, FL
